# Practical Notes and Formulæ

**Published:** 1883-07-20

**Authors:** 


### PRACTICAL NOTES AND FORMULÆ.

**Cathartic for Infants.**—Dr. J. Cooperider, Taylorsville, Indiana, sends in response to the request for a cathartic for infants, the following which he has found quite satisfactory in his practice:

℞ Ext. sennæ fluidt ............................ ℥j,

Sodii sulphatis ............................ ℥ss.

Spts. limonis ............................ ℥j,

Syrupi. q. s., ad ............................ ℥iv.

M. Sig. A half teaspoonful to a teaspoonful every two to four hours, according to age.

Dr. M. R. Morden, Adrian, Mich.: In answer to Dr. Postlewait, in regard to a suitable remedy for constipation in infancy, I wish to attest my confidence in the pulvis glycyrrhizæ compositus for the same. It consists of:

℞ Senna ............................ }

Liquorice (powder) ............................ } aa ʒ vj,

Fennel ............................ }

Sulphur ............................ } aa ʒ iij,

Refined sugar ............................ ʒ xviij.

May be given best in form of a thick tea, in one-quarter to one-half teaspoonful doses to infants, and to large children, aged persons and delicate adults in teaspoonful doses, once, twice or thrice daily, as may be needed. After 10 years' experience with its use I recommend it as being pleasant, safe and efficient. One dose at bed-time generally answers. I never knew of but one child that did not like it.

Dr. Wm. R. Smith, Sr., Cairo, Illinois, writes: From my own experience this winter, I think the constipation of the little patients, refeıred to by Dr. Postlewait, is caused by catarrh of the colon and rectum. Would advise him to give the following:

℞ Sodii phosphatis granulati ............................ ʒ ij,

Ft. chart ............................ NO. viij.

Sig. One powder in a tablespoonful of milk three times daily.—*Med. Age.*

**Aloes Externally.**—"If aloes be applied to an ulcer or excoriated surface, it will act as a brisk purgative, producing stools of the same kind, as if administered internally."—Warner. "One part tinct. of aloes and two of soap liniment rubbed for five or ten minutes daily over the abdomen of infants of habitual constipation very effectually keep the bowels open."—Merriman.—*Ex.*

**Tannate of Cannabine.**—A few months since M. Fronmuller presented tannate of cannabine as a very efficient medicament to induce sleep without any disagreeable after-effects. The dangers of the abuse of morphine are so great that any other agent capable of inducing similar hypnotic effects would be a veritable boon to the practitioner.

Mr. Hiller has experimented with the medicament, and has found that it gives good results, particularly in the milder forms of sleeplessness. Its effects are less marked in serious cases of delirium tremens, in mania and in subjects already habituated to narcotics. It may be ordered in powders:

℞ Tannate of cannabine.......................... gr. xv,
Sacch. alb.................................. gr. xx.
M. et divid. in cht. No. iv.

Sig. One or more at bed-time.—*Med. and Surg. Rep.*

**Grindelia Robusta in Asthma.**—

℞ Fl. ext. grindelia rab.............................. ℥ j,

Fl. ext. yerba santa.................................. ℥ ss,

Simple syrup.......................................... ℥ ss.

M. Sig. Take one teaspoonful of this every three or four hours during the day and oftener at night. He was relieved considerably very soon, and after continuing this for over a week I made him a prescription of—

℞ Fl. ext. grindelia rob.............................. ℥ j,

Fl. ext. belladonna................................... ʒ ij,

Iodide of potassium................................... ʒ ij,

Simple syrup.......................................... ℥ j.

M. Sig. One-half teaspoonful every four hours, and to be increased if necessary.—*Thera. Gaz.*

**Eczema.**—Dr. Duhring's favorite prescriptions for eczema are:

℞ Carbolic acid,

Glycerine,

Alcohol.............................. aa fl. ʒ iv,

Water.................................... O j.

M. Wash the parts with this thoroughly, and then rub in well.

℞ Sulphur ointment,
Tar ointment,
Mercury ammoniated ointment.......... aa ℥ ss.

—*Medical Herald.*

**Clergyman's Sore Throat.**—Dr. Springstein, in the Medical Brief, recommends the following as a useful palliative, and, in some cases, a cure for this troublesome disease:

℞ Tinct. opii.,

Tinct. sanguinariæ.......................... aa fl. ℥ ij,

Balsam tolu...................................... ʒ ij.

M. Sig. Twelve drops on a lump of sugar 3 or 4 times a day. —*Boston Jour. Chem.*

**Kalsomining Fluid.**—Some of our readers may be interested about this season of the year in such information as is given in the following, which we clip from the Weekly Drug News and American Pharmacist, which commends it as a good kalsomining fiuid for walls:

| | |
|---|---|
| White glue | 1 pound, |
| White zinc | 10 pounds |
| Paris white | 5 pounds, |
| Water | sufficient. |

Soak the glue over night in three quarts of water, then add as much water again, and heat on a water-bath till the glue is dissolved. In another pail put the two powders and pour on hot water, stirring all the time, until the liquid appears like thick milk. Mingle the two liquids together, stir thoroughly, and apply to the walls with a whitewash brush.—*Med. Age.*

**A New Invisible Ink.**—A new method of making an invisible ink has been discovered, the ingredients of which are:

| | |
|---|---|
| Linseed oil | 1 part, |
| Ammonia water | 20 parts, |
| Water | 100 parts. |

To use this ink the pen is dipped in the ink and the secret is written invisibly on paper. When the paper is dipped in water, and while it is wet, the secret can be read. As the paper dries the secret again disappears.—*Jour. of Health.*

**Treatment of Consumption by Causing them to Breathe an Antiseptic Atmosphere.**—Schueler and Griefswald, found that animals rendered artificially tuberculous, were cured by being made to inhale vaporized creasote-water for lengthened periods of time. The opinion is rational, because by continued inhalation alone can the affected portions of the lung be so reached as to be benefitted by the medicine employed.—*Ex.*

**A Palatable Cough Mixture.**—The most elegant and palatable cough mixture ever prescribed by Dr. J. Milner Fothergill, is, he says, the following:

| | |
|---|---|
| ℞ Syr. scillæ | ʒ j, |
| Acid. hydrobromic dil | ʒ ss, |
| Spts. chloroform | ʒ ss, |
| Aquæ | ℥ j. |

—*Medical Summary.*

**Bleeding Piles.**—

| | |
|---|---|
| ℞ Spec. tinct. hamamelis | ℥ j, |
| Spec. tinct. collinsoniæ | ℥ ss, |
| Spec. tinct. apocynum can | ℥ ij, |
| Syr. simp. q. s., | ℥ iv. |

M. Sig. Teaspoonful every four hours.—*Med. Brief.*

